# Identifying the Effects of Social Disruption through Translocation on African Elephants (*Loxodonta africana*), with Specifics on the Social and Ecological Impacts of Orphaning

**DOI:** 10.3390/ani13030483

**Published:** 2023-01-30

**Authors:** Marion E. Garaï, Victoria L. Boult, Heike R. Zitzer

**Affiliations:** 1 Elephant Reintegration Trust, Port Alfred 6170, South Africa; 2 Department of Meteorology, University of Reading, Reading RG6 7BE, UK

**Keywords:** African elephants, social disruption, orphans, calf mortality, reproduction, social competence, leadership, cohesiveness, anthropogenic effects

## Abstract

**Simple Summary:**

The translocation of elephants is a management tool developed in the 1980s during the culling operations at the Kruger National Park, South Africa, to remove “surplus” elephants from fenced properties. Elephants live in large social networks and form strong social bonds within their family units. In particular, the mother–offspring bond is crucial to the learning and development of social skills and social and environmental competence of the calves. The leadership role and experience of the matriarch appear to be an important factor in providing the necessary knowledge to optimise social and environmental skills and competence. The translocation of smaller groups of elephants results in the social disruption of these networks. This paper looks at the social and ecological aspects of such disruption and what it implies for elephants. A herd of Orphans and a translocated herd consisting of two families were observed over several years. The Orphans demonstrated marked effects of social disruption by splitting more frequently and for longer periods than the family herd and experiencing accelerated reproduction. Social disruption may therefore reduce learning opportunities with implications for elephant society as well as for conservation.

**Abstract:**

African elephants (*Loxodonta africana*) exhibit a long developmental period during which they acquire complex social and ecological knowledge through social networks. Central to this is that matriarchs and older individuals play an important role as repositories of information gained through experience. Anthropogenic interventions—including poaching, culling, translocation, and hunting—can disrupt elephants’ social networks, with implications for individual fitness and potential long-term population viability. Here, we draw on a unique long-running, individual-based dataset to examine the impacts of translocation on a population of elephants in South Africa, taking into consideration demographic rates, social dynamics, and ecological decision-making. Specifically, we compared two translocated groups: a group of unrelated culling Orphans and a family herd. We found that the Orphan group experienced accelerated reproductive rates when compared with the family herd. The Orphan group also fissioned more frequently and for longer periods of time, suggesting lower cohesiveness, and were less decisive in their large-scale movement decisions. These results add to the growing body of literature on the downstream impacts of social disruption for elephants. Whilst the translocation of culling Orphans is no longer practised in South Africa, we encourage careful consideration of any elephant translocation and the resulting social disruption.

## 1. Introduction

Sociality enables social learning (learning that is influenced by observation of, or interaction with, another conspecific individual [1]), which is the basis of developing social competence [2] to optimise social relationships [3], develop coping strategies [4], and acquire ecological competence [5], which in turn helps optimise foraging, landscape use, health [6], and ultimately fitness. In some species, individuals with strong and consistent social ties live longer [7,8] and enjoy higher reproductive success [9]. Intact social environments are especially important for cognitively advanced mammals, which live in complex societies and form lifelong bonds, including elephants [10], cetaceans [11], and non-human primates [12].

Elephants have a long calf and adolescent development period [13], during which they must accumulate environmental and social knowledge [14], acquire skills in communication [15], tool use [16], and food finding and preparation [17]. These skills are obtained through social learning [1,18] amongst kin and via social networks [19], whereby growing up without the leadership of mothers and matriarchs may have important consequences for survival and reproduction and may negatively impact population dynamics, fitness [20], and ultimately elephant conservation [21].

Here, we consider how social disruption impacts social and ecological decision-making in a population of elephants in South Africa. We define social disruption as any breakage of close social bonds between mothers and their offspring, closely related kin, or larger social networks. Social disruption in elephants can occur through anthropogenic interventions such as poaching [22,23], culling, hunting, and translocation [24]. Elephants, having experienced such events, have also been shown to lack social competence [21], decision-making skills [24], calf-rearing abilities, and environmental knowledge [25].

Particular events likely to impact an individual’s social ability are early-life trauma, the loss of matriarchs—who act as repositories of social and environmental knowledge [18,26,27]—and inexperience [28], or loss of a mother or other older female relative [29]. Socially learned behaviour can be crucial to how animals interact with each other and the environment, and loss thereof can allow the spread of maladaptive behaviour [30]. Loss of social competence through early negative experience has been described for chimpanzees [31], and in other species, females who experienced more cumulative early adversity have significantly shorter lifespans [32]. Additionally, adverse events at an early age can have intergenerational effects [33,34], with a female’s early life environment impacting many physiological and behavioural aspects of her offspring, such as disease, reproductive rates and success, development, fitness, foraging strategies, stress response, personality, and body size [33,35,36].

Asian elephants growing up without their mothers and families not only exhibit abnormal behaviours, an impaired knowledge of food preparation, and an inability to socially integrate but are also more likely to experience stunted growth, short pregnancies, and low offspring birth weight [17]. In African elephants, the lack of older, experienced matriarchs has been seen to induce higher mortality rates for the remaining members of the herd [28].

The translocation of family groups and individual elephants has long been used as a management tool in South Africa to prevent habitat over-utilisation without having to cull [37]. Translocation can disrupt social networks [21], thereby severely impacting an individual’s opportunities for learning. Further, societal breakdown caused by translocation may induce aberrant behaviour akin to post-traumatic stress disorder in humans [29,38].

Owing to the near extinction of elephants across South Africa during the 19th and 20th centuries, most elephant populations in fenced reserves in South Africa originate from translocated individuals, largely from the Kruger National Park (KNP) [39]. Early translocations during the 1980s and early 1990s typically moved groups of young, often unrelated elephants orphaned during KNP’s culling operations. Later, due to ethical concerns and advances in logistical capabilities, cow herds were translocated in their entirety. Translocated groups of varying sizes and origins, some as small as four elephants [40], thus formed the founding members of elephant populations in fenced reserves across the country. In South Africa, the National Norms and Standards for the Management of Elephants (2018) [41] are based on guiding principles, which, amongst others, state that disruption of social groups as a result of management intervention should be avoided or minimised. However, little was known of the consequences of such management interventions, and to this day, the unintended long-term negative consequences on the lives of the elephants [42] are rarely considered in management decisions.

The Pongola Game Reserve (PGR) in KwaZulu-Natal provided the unique opportunity to compare the impacts of different methods of translocation and consider the long-term consequences of social disruption caused by translocation. The reserve is originally home to three translocated herds: a group of culling orphans and their offspring (hereafter, the “Orphans”) and two family groups, which merged (hereafter the “A&B herd”). Although the Orphans are now adults with their own offspring, we hypothesised that they would display more signs of social disruption when compared with the A&B herd due to the additional disruption and social breakdown caused by their orphaning before translocation. We, therefore, predicted the following:

1. The Orphans would be less competent in rearing their young than the A&B herd, demonstrated by a younger average age of death and a higher calf mortality rate [26,43,44], and we expected this to be reflected in rates of vigilance behaviour, with the Orphans exhibiting less awareness of danger and the appropriate response;
2. Except for possibly two of the founding members, the Orphans are not thought to be kin and are instead remnants of different herds pieced together for translocation from KNP. We, therefore, expected the Orphans to be less cohesive as a unit, causing the group to fission more frequently and exhibit less affiliative behaviour with herd mates (and also because they were not exposed to such behaviours by their own mothers and allomothers);
3. Previous research has linked early adversity with accelerated reproductive development in human and non-human females [45,46,47,48]. We, therefore, expected the Orphans to have shorter inter-birth intervals (IBI) than the A&B herd and to commence reproduction at a younger age (Garaï pers. obs.);
4. Despite the founding members of the Orphans all being similar in age, as noted in other orphaned elephant groups [49], we expected one individual to assume the role of matriarch. However, whilst matriarchs typically act as a vital repository of social and ecological knowledge, we assumed that the Orphans’ matriarch would lack much of this knowledge due to early-age orphaning and thus no access to older females to learn from example. We expected this to play out as a less-definitive leadership approach demonstrated through her position relative to her herd and the decisiveness of herd movement decisions.

## 2. Methods and Material

### 2.1. History of the PGR Elephants

**Figure 1:**
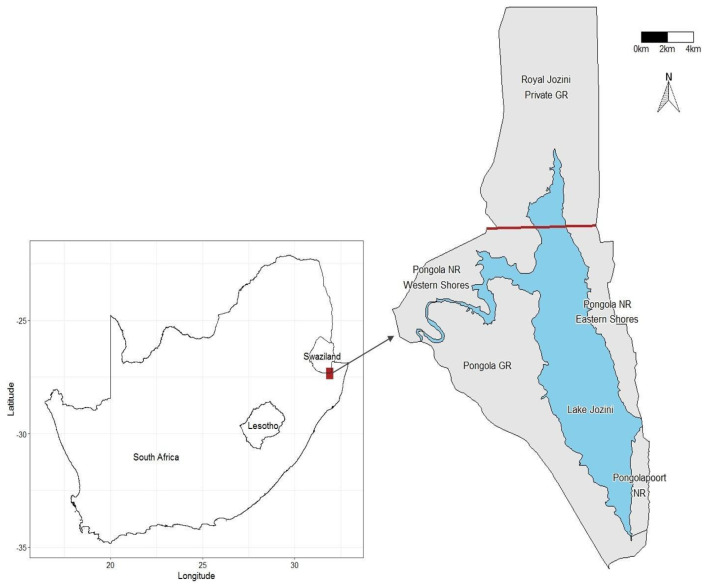
The geographical arrangement of reserves comprises the Pongolapoort ecosystem. GR = Game Reserve (private). NR = Nature Reserve (provincial). The bold dark red line indicates the international border between South Africa and Eswatini (formerly Swaziland). Dubula is a private section west of the Pongola NR Western Shores.

The Pongola Game Reserve (PGR) is a small (~10,000 hectares) [50] private reserve situated in northern KwaZulu-Natal, South Africa (Figure 1). PGR is fenced at the south and west and bounded by the Pongola River to the north and Lake Jozini to the east. The lake straddles the international border between South Africa and Eswatini and is surrounded by a matrix of private and provincial reserves.

A number of elephant groups and individuals were translocated to PGR between 1997 and 2001. Prior to these translocations, elephants had been absent from the region for many decades. In 1997, two family groups, the A (n = 8) and B (n = 9) families, were translocated from KNP to PGR. Although separately translocated, the two-family groups soon formed a single herd, thereafter named the A&B herd. Three adult males were introduced from KNP in 1998. A group (n = 5) of unrelated, subadult elephants (aged between 10–13 years), orphaned in 1992 during the KNP culling operations, were originally translocated to a nearby reserve but, guided by one of PGR’s bulls, moved into PGR in 2000, thereafter known as the Orphans. The Orphans attempted to assimilate with the A&B herd but were not accepted, so they were treated as their own group. Furthermore, three adult males originally translocated from KNP also moved into PGR from a nearby reserve in 2001, bringing the founding PGR elephant population to 28 individuals (Table 1).

Although initially arriving on the PGR, the elephants have subsequently moved across the Pongola River and around Lake Jozini into neighbouring private and provincial reserves (Figure 1). The movement of elephants into other reserves was facilitated by the falling water levels of Lake Jozini following several years of below-average rainfall and continued agricultural offtake; elephants moved around boundary fences and through the river in the shallow water. Such exploration and movement are natural for elephants, but we believe it may have been further provoked by dry conditions and declining vegetation on the PGR [51].

Whilst in this paper we seek to understand the effects of translocation on female elephant groups, it should be noted that the PGR elephant population has undergone additional stressors which may add to atypical behaviours [52]. Management interventions intended to minimise elephants’ population growth, limit elephant impacts on vegetation, and guard human safety have included:

- Vasectomising seven subadult bulls in 2008;
- GnRH contraceptive treatment of the two oldest bulls (unable to undergo vasectomies) from 2008 until their deaths in 2010 and 2013;
- Destruction of a dominant, mature A&B herd cow in 2011;
- GnRH treatment of five subadult bulls from 2016–2017 as a means of contraception
- Hunting of adult bulls in 2010 and 2013;
- Forced movement of the A&B herd from the Royal Jozini Private Game Reserve (RJ) to the Pongola Nature Reserve Eastern Shores on 11 October 2016, during which time the herd was split, and a subset of the A&B herd (n = 7) remained in RJ. This separation persisted until the end of the study period.

### 2.2. Data Collection

#### Demographic and Behavioural Monitoring

The PGR elephant population was intensively monitored from February 2011 to December 2018. Owing to the separation of the A&B herd on 11 October 2016 and the additional social disruption caused, we chose to exclude data collected after this time and solely focus on the impacts of translocation. All data were collected by Heike Zitzer or by others under her supervision to ensure data were consistent and accurate. Monitoring took place for 6–8 h most days. The elephants were monitored routinely from a vehicle, although ad hoc observations made on foot or via camera traps were also included in the dataset. All efforts were made to minimise the influence of the observer on elephant behaviour: the same monitoring vehicle was used whenever possible and was positioned within the elephants’ field of vision but at a minimum distance of 80 m, allowing the elephants’ acknowledgement without obstructing their intended movement paths or intruding into their comfort zones.

Six adult males and the matriarch of the Orphans (Constant) were fitted with VHF radio collars to facilitate locating the elephants each day. No member of the A&B herd was collared for any meaningful length of time during the study period. Instead, the A&B herd was located based on the elephant monitor’s deep knowledge of their habits and movement patterns. All effort was made to monitor each herd and all individuals regularly and equally. If one individual or herd was not observed for a period of time, this was because they were inaccessible. Upon locating elephants, two sampling techniques were employed to monitor their behaviour (a full ethogram is included in the Supplementary Materials):

**Instantaneous 30 min scan samples [53]** recorded the time, date, and location of the elephant(s) being observed. The number of elephants was counted, and any focal individuals were identified (based on unique ear tears and tusk shape). The habitat type and dominant vegetation species were recorded. Finally, the behaviour displayed by the majority of the group at the time of the scan sample was recorded as either feeding, resting, moving, or other.

**Continuous 15 min focal samples** monitored a single focal elephant continuously for 15 min (or for as long as the individual was visible if less than 15 min). These were completed between subsequent 30 min scan samples. During this time, all behaviours exhibited by the focal individual were recorded, along with the time that behaviours began and ceased, and any interactions with other individuals were noted. Vigilance behaviours (head held high, tail raised, ears lifted and held outward at an angle and looking specifically in one direction) were recorded in order to determine each herd’s response to possible threats. Affiliative behaviours (body contact, play, greetings, and genital inspections) were recorded to examine the strength of relationships between individuals. This position of the focal individual relative to the rest of the herd was also noted, as this is representative of their role within the group; matriarchs typically occupy positions either leading the herd from the front, moving on the outer edge of the herd, or following the herd, though other females may also occupy these positions and, in doing so, display leadership tendencies [54].

Any demographic events (new births or deaths) were recorded when they were realised, but dates were usually accurate to a couple of weeks, given the frequency of monitoring. Group size and composition at the end of the study period are shown in Table 2.

### 2.3. Data Analyses

All behavioural data were converted to the proportion of total observation time or count in order to adjust for differences in total observations of each group or focal individual. Instantaneous scan samples provided information on space use and fission–fusion dynamics of both groups, whilst continuous focal samples recorded the amount of time spent exhibiting affiliative and vigilance behaviours. Given that affiliative behaviours may be more common amongst a larger group and vigilance may be heightened in a smaller group, we further standardised the proportion of time spent exhibiting vigilance and affiliative behaviours by group size.

Demographic data were compared between the two groups. Infant mortality rate (≤2 years old) and sex ratios at birth were calculated only for individuals born after arrival at PGR. Females suspected of previously having a calf before arrival in PGR were excluded from calculating the mean age of their first parturition. Inter-birth intervals (IBI) were only calculated for multiparous females. It is possible that some births went unrecorded, particularly stillbirths or deaths occurring soon after birth. However, the close monitoring of PGR elephants means that pregnancies were often suspected before parturition, and behavioural anomalies could be used as an indicator of stillbirth or calf death.

We statistically compared differences in mean behavioural and demographic rates between the A&B herd and the Orphans. Most data were found to violate the assumption of normality, and the transformation of the data was unsuccessful. Where this was the case, non-parametric tests were used (detailed in the Results below).

## 3. Results

### 3.1. Calf-Rearing Ability

#### Age of Death and Infant Mortality

A lack of older, experienced females suggests that the Orphans should experience higher infant mortality rates, pulling down the average age of death. Further, it was expected that more infants would die in the circumstances suggesting a lack of experience by their mothers (e.g., being hit by a train, predation, or drowning).

**Figure 2:**
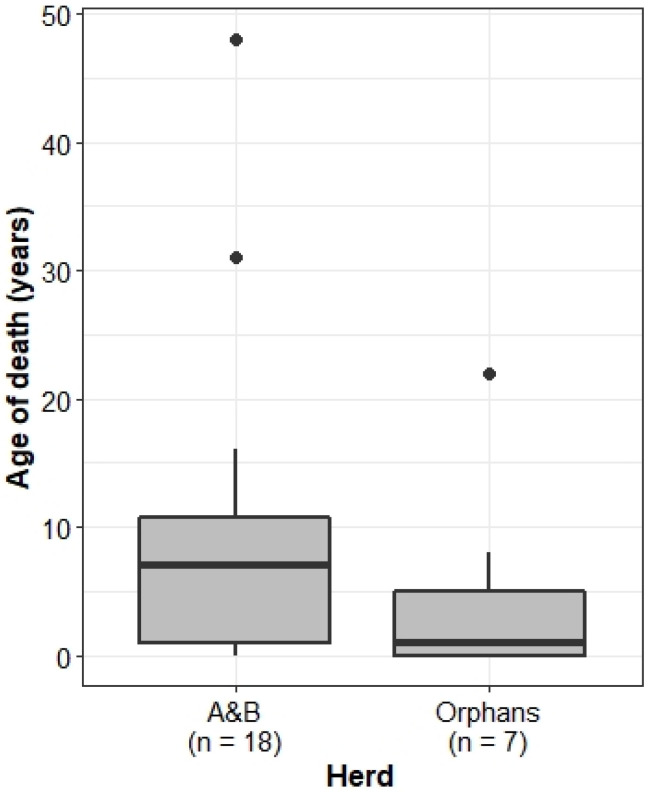
Age of death of individuals in the A&B herd and Orphans. Boxplots show median, IQR, and outliers. Numbers on the *x*-axis indicate the number of deaths recorded in each herd.

Whilst the median age of death was lower for the Orphans (1 year old) than the A&B herd (7 years old), this difference was not significant (Wilcoxon rank sum test: W = 84.5, *p* = 0.1986) (Figure 2). Of all calves born to the Orphans, 21% died ≤2 years of age (n = 5 of 24), whilst in the A&B herd, only 9% of individuals died as infants (n = 7 of 78), although these differences were not significantly different from expected values (Chi-squared test: X^2^ = 1.4305, df = 1, *p* = 0.2317).

Of these infant mortalities, the Orphans lost more infants to the train (n = 2, accounting for 40% of infant mortalities) than the A&B herd (n = 1, 14% of infant mortalities), but the A&B herd additionally lost three infants to drowning (accounting for 43% of infant mortalities).

### 3.2. Vigilance Behaviour

**Figure 3:**
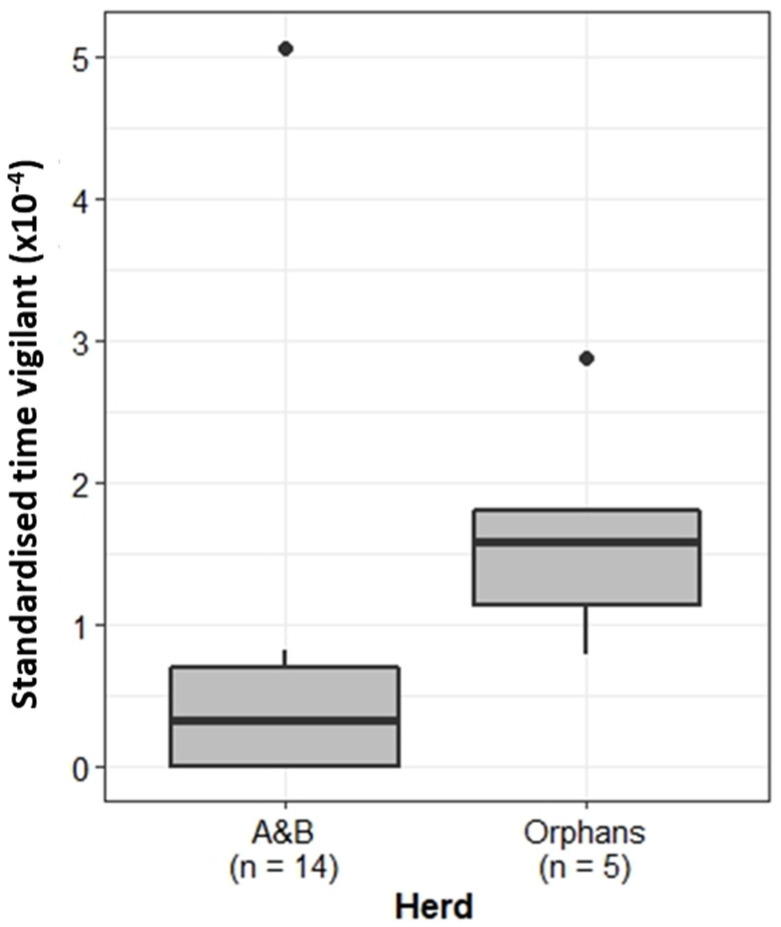
Proportion of time the A&B herd and Orphans spent exhibiting vigilance behaviour. Boxplots show median, IQR, and outliers. Numbers on the *x*-axis indicate the number of individuals included in the analysis for each herd.

Vigilance is important to identify and correctly respond to potential threats. Comparison of the proportion of time focal females spent displaying vigilance behaviour showed that, when standardised by group size, individuals belonging to the Orphans displayed significantly more vigilance behaviour than the A&B herd (Wilcoxon rank sum test: W = 6, *p* < 0.01) (Figure 3).

### 3.3. Cohesiveness

#### 3.3.1. Fission–Fusion Dynamics

Given that most of the founding members of the Orphans are not thought to be closely related (with the possible exception of two adult females), it was expected that the Orphans would be less cohesive than the A&B herd. A less cohesive herd is expected to fission more frequently, spending time as subunits of the entire herd [55] (205–223).

**Figure 4:**
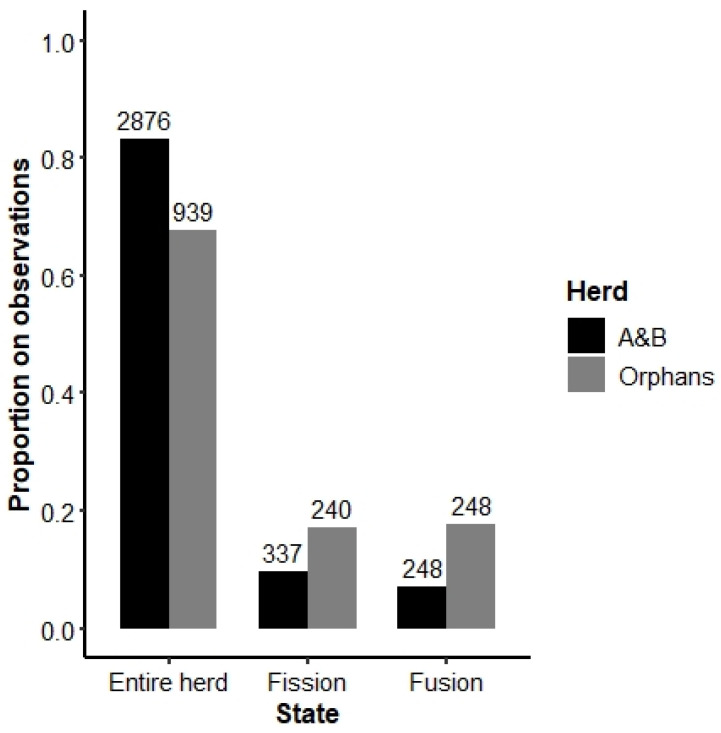
Fission–fusion dynamics of the A&B herd and the Orphans. “Entire herd” refers to observations in which all members of either the A&B or Orphans herd were present. “Fission” indicates instances in which only a subsection of either the A&B herd or Orphans were observed. “Fusion” refers to instances in which all or some members of both the A&B herd and Orphans were present. Numbers on top of the bars indicate the number of scans included in each category.

As expected, the Orphans spent proportionally less time as an entire herd and more time fissioned than the A&B herd (Chi-squared test: X^2^ = 72.73, df = 1, *p* < 0.001), suggesting that the Orphans are indeed less cohesive (Figure 4). Additionally, Orphan members spent proportionally more time fused with the A&B herd (Chi-squared test: X^2^ = 140.51, df = 1, *p* < 0.001), perhaps suggesting that the inexperienced Orphans were seeking the guidance of older females in the A&B herd.

**Figure 5:**
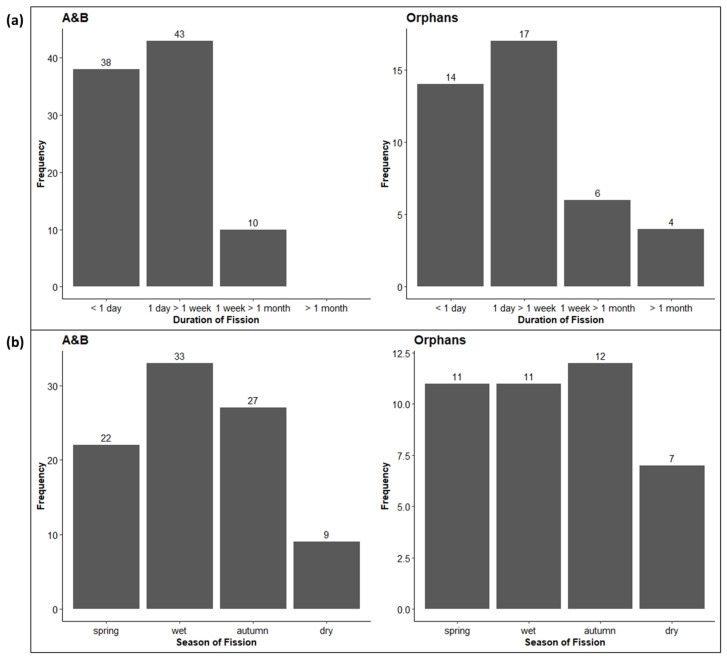
(**a**) The duration of fission events for the A&B herd and the Orphans. (**b**) The seasonality of fission events throughout the year. Numbers above bars indicate the number of scans in each category. Seasons: spring = September–October, wet = November–February, autumn = March–May, and dry = June–August.

The duration of fission events was significantly longer for the Orphans (median 3086 min or nearly 3 days) than for the A&B herd (median 1633 min or just over a day) (Chi-squared test: X^2^ = 37.73, df = 3, *p* < 0.001), but considering the distribution of the duration of fission events (Figure 5a), the pattern is similar for both herds with frequent short-term fissions most commonly lasting between 1 day and 1 week. However, the A&B herd never fissioned for more than a month, whilst the Orphans did on several occasions. Seasonally, fission events did not uniformly occur throughout the year (Figure 5b), with the A&B herd fissioning most often in the wet season and the Orphans fissioning most often in autumn. However, the seasonal differences were not significant (Chi-squared test: X^2^ = 2.04, df = 3, *p* = 0.56).

#### 3.3.2. Affiliative Behaviour

**Figure 6:**
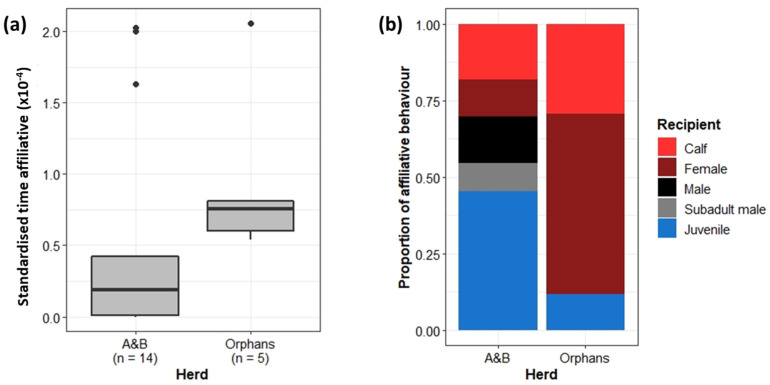
(**a**) Proportion of time spent exhibiting affiliative behaviours in the A&B herd and Orphans. Boxplots show median, IQR, and outliers. Numbers on the x-axis indicate the number of individuals included in the analysis for each herd. (**b**) The proportion of affiliative behaviour directed towards other demographic groups. “Male” refers to independent adult bulls. “Subadult male” refers to semi-independent sub-adult bulls.

The less cohesive Orphans were also expected to demonstrate less affiliative behaviours towards one another as social bonds were assumed to be weaker. However, when standardised by group size, the Orphans spent significantly more time exhibiting affiliative behaviours than the A&B herd (Wilcoxon rank sum test: W = 12, *p* < 0.05) (Figure 6a). Further, the recipients of affiliative behaviours significantly differed between the A&B herd and the Orphans (Chi-squared test: X^2^ = 17.25, df = 4, *p* < 0.01). The Orphans directed a higher proportion of affiliative behaviour towards herd mates (other adult females, calves, and juveniles within their herd) than the A&B herd, who rather directed affiliative behaviour more often towards males (Figure 6b).

### 3.4. Accelerated Reproduction

#### 3.4.1. Age at First Parturition

**Figure 7:**
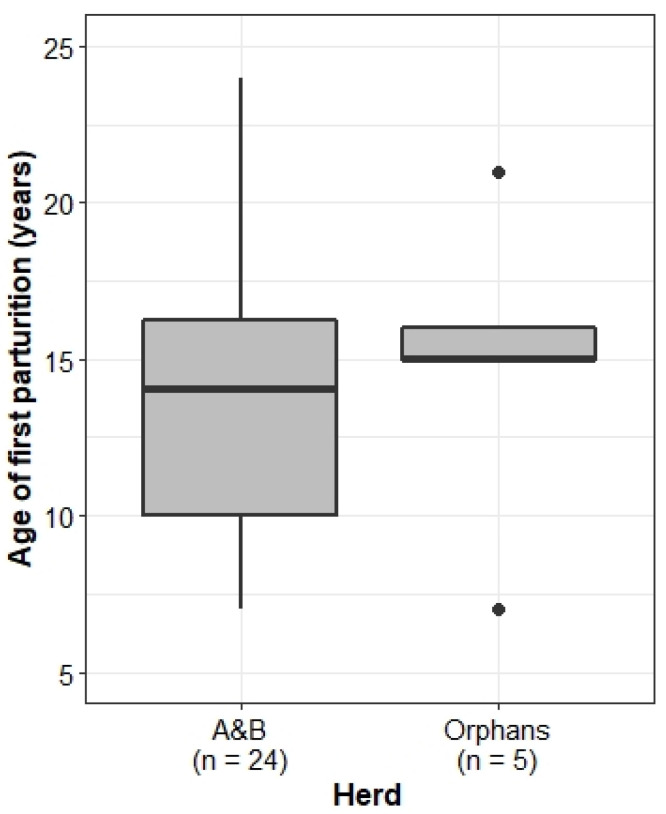
Age of the first parturition of females in the A&B herd and the Orphans. Boxplots show median, IQR, and outliers. Numbers on the *x*-axis indicate the number of individuals included in the analysis for each herd.

Socially disrupted humans and non-human mammals have been observed to experience accelerated reproductive rates, so we expected the Orphans to mature earlier and give birth more frequently than the A&B herd. Comparison of the age of the first parturition, however, showed no significant difference between the two herds (Welch’s two-sample *t*-test: *t* = −0.31, df = 5.5, *p* = 0.77) (Figure 7). 

#### 3.4.2. Inter-Birth Intervals

**Figure 8:**
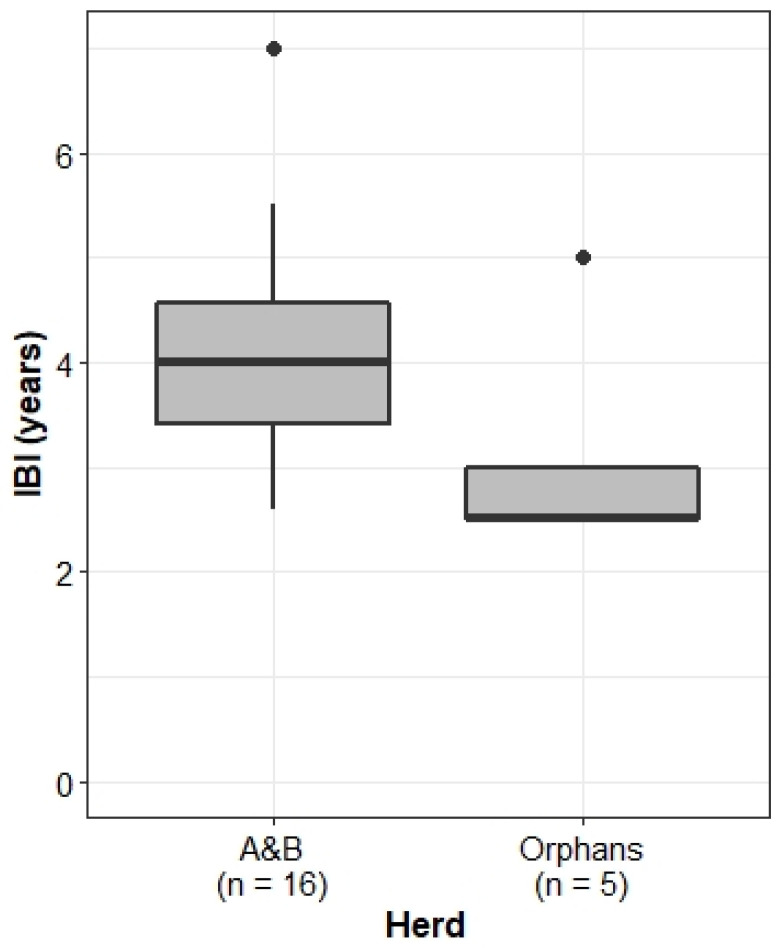
IBI for multiparous females in the A&B herd and the Orphans. Boxplots show median, IQR, and outliers. Numbers on the *x*-axis indicate the number of individuals included in the analysis for each herd.

The Orphans did, however, exhibit significantly shorter inter-birth intervals than the A&B herd (Wilcoxon rank sum test: W = 64.5, *p*-value < 0.05; Figure 8).

We considered a number of reasons for the differences in IBIs. Moss & Lee [56] (187–204) found that higher infant mortality rates led to shorter IBIs in the Amboseli elephant population. However, we see no significant difference in infant mortality rates between the A&B herd and the Orphans (Figure 2). Physiologically, the energetic demand of calf-rearing on mothers presents a potentially limiting factor in calf survival. Younger mothers tend to be smaller, with fewer energy reserves than older females [28,57]. In addition, due to greater birth weights and faster growth rates, male calves pose a larger energetic demand on their mothers than female calves [57,58]. Whilst there was no significant difference in the mean age of first parturition between the Orphans and the A&B herd (Figure 7), there was a dramatic difference in calf sex ratios. Natural elephant sex ratios at birth are expected to be 1:1 [56]. Whilst the A&B herd maintained a near 1:1 calf sex ratio (51% male), the sex ratio of the Orphans was dramatically skewed, with all but one calf being male (92%).

#### 3.4.3. Sex Ratios

**Figure 9:**
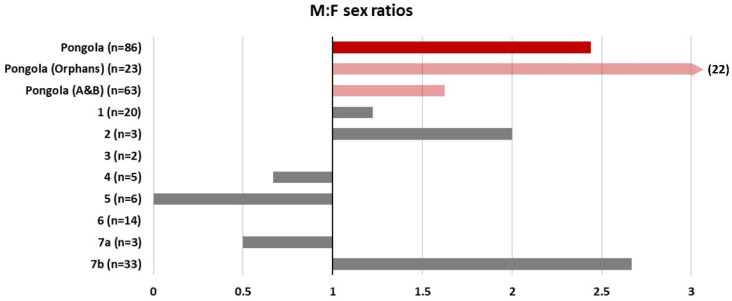
Sex ratios at birth for a number of socially disrupted elephant populations across South Africa. Reserve names have been anonymised. Pongola is shown in red, and the two herds are separately shown in pink. All other reserves are shown in grey. Note that the sex ratios of calves born to the Orphans extend beyond the bounds of the plot. Reserve 5 consists of a group of ex-trained elephants, and 7a and 7b are originally one group split by management within the same reserve.

The Orphans’ skewed sex ratio led us to compare with other reserves that are home to socially disrupted elephant populations. Although the mean sex ratio across the reserves did not significantly differ from the expected 1:1 ratio (one-sampled *t*-test: *t* = 0.92, *p* = 0.39), it does appear that in a number of reserves, there is a trend towards more males being born (Figure 9), and only two reserves had the expected 1:1 sex ratio.

### 3.5. Decision Making

#### 3.5.1. Leadership

With no multigenerational age structure and all adult females being of a similar age, it was unclear which member of the Orphans would be the matriarch. Through close observation over many years, Constant (female born approx. in 1987) seemingly fulfilled the role of matriarch. However, given the non-natural age composition of the Orphans, it was expected that Constant’s leadership would be less dominant than Antares’ (matriarch of the A&B herd, born approx. in 1963). The spatial position of matriarchs in relation to the rest of their respective herds was compared to assess this. Matriarchs typically occupy positions either leading the herd from the front, moving on the outer edge of the herd, or following the herd [54]. However, other females may also occupy these positions and, in doing so, display leadership tendencies. Other females of the Orphans were expected to occupy these locations more frequently than adult females in the A&B herd.

**Figure 10:**
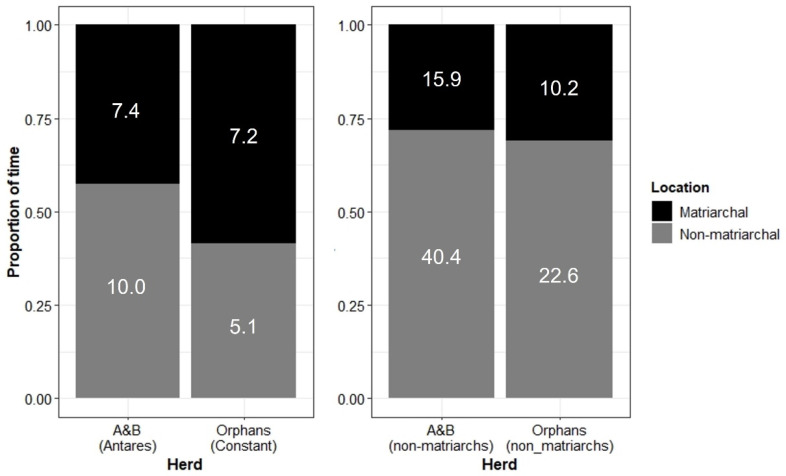
(**Left**): The position of matriarchs Antares (A&B herd) and Constant (Orphans) in relation to the herd. (**Right**): The position of non-matriarch adult females in relation to the herd. “Matriarchal” positions include following, leading (matriarchs)/or moving at the front (non-matriarchs), or moving on the outer edge of the herd. “Non-matriarchal” positions include moving within the herd, following at the rear/trailing the herd, or not with the herd (see Supplementary Materials for full ethogram). Numbers within bars show the number of focal hours contributing to each category.

Contrary to expectations, however, focal females of the Orphans spent a similar proportion of time in matriarchal positions to focal females in the A&B herd. Interestingly, Constant (the Orphans’ matriarch) actually spent more time in matriarchal positions than Antares (the A&B herd’s matriarch) (Figure 10).

#### 3.5.2. Space Use

**Figure 11:**
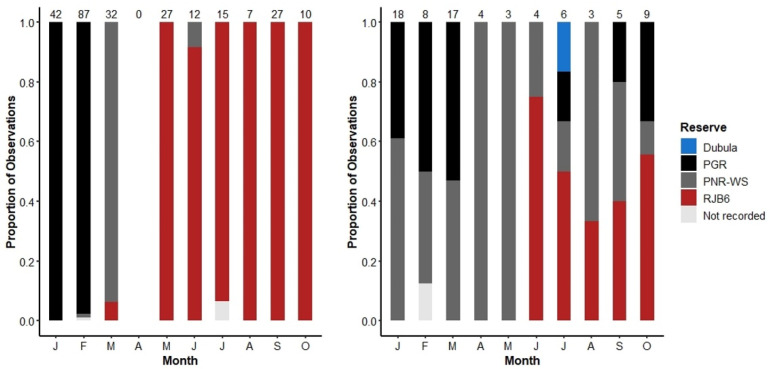
The movement of the A&B herd (**Left**) and Orphans (**Right**) during 2016. Bars indicate the proportion of observations in each reserve. For a map of the reserves, see Figure 1 and refer to the Methods section. Note that no observations were made of the A&B herd during April 2016.

The Orphans demonstrated different movement patterns to the A&B herd during the move into the Royal Jozini Private Game Reserve (RJ), Eswatini, in 2016 (Figure 1). The A&B herd demonstrated purposeful and directional movement first from PGR to the Pongola Nature Reserve Western Shores (PNR-WS) and then onto RJ, all within the span of 3 months (Figure 11). The A&B herd remained at RJ almost exclusively until the main part of the A&B herd was pushed to the Pongola Nature Reserve Eastern Shores (PNR-ES) on 11 October 2016 (data excluded from this analysis). Note that there were no recordings of the A&B herd in April 2016 because the elephant monitor could not reach RJ.

By contrast, the Orphans only moved onto RJ in June 2016, and even then, they were less committed, continuing to divide their time between RJ, PGR, PNR-WS, and Dubula (Figure 1).

It should be noted that the scan data does not provide a perfect representation of herd movements as it was somewhat limited by the ability of the monitor to access reserves and locate herds. However, every effort was made to monitor all reserves in each month, and so an absence of records in a given reserve in a given month is a strong suggestion that the herd was not present.

## 4. Discussion

Highly social and cognitive animals, such as elephants, optimise fitness via social learning between mothers and their offspring, closely related kin, or larger social networks. Disruption and breakdown of bonds and social networks through management interventions, such as translocation, will have negative effects [1,2,10,19,24,59]. We had the opportunity to observe two herds of translocated elephants—a family herd originally consisting of two family units and an orphaned herd—to assess the long-term impacts on social competence and, thus, their ability to optimise fitness.

### 4.1. Calf Rearing Ability

Social disruption has been found to have negative impacts on elephant mortality and reproductive success [21,60]. Lack of early social attachment and care from the mother and family could also affect calf development of normal emotional and social abilities [61], with the effects being passed on to the next generation [62]. We expected that orphaning early in life would result in the Orphans having a limited ability to correctly identify and respond to danger, which would, in turn, lead to an earlier age of death of their offspring. Whilst the average age of death did not significantly differ between the two herds, the Orphans displayed significantly more vigilance and ignorance of danger than the A&B herd. This is possibly an example in which the Orphans overcompensated, being unnecessarily alerted by stimuli, as they had not learnt from a matriarch what events constituted real danger and therefore made them more nervous. The incorrect direction of vigilance is further supported by the fact that the Orphans lost more calves to the train than the A&B herd, suggesting an inappropriate recognition of, or response to, the threat posed by the train. Anecdotally, the A&B herd chose to cross the railway in only a few locations throughout the reserve where visibility of the oncoming train was greatest, whilst the Orphans crossed the railway more freely. Throughout the study, we observed that the A&B herd was more often seen on floodplains in close proximity to Lake Jozini, potentially explaining the higher number of calves dying due to drowning.

Whilst the Orphans displayed more vigilance behaviour relative to group size, vigilance behaviour was actually comparatively rare across the dataset. This could be explained by social disruption, as we posit here, but it is also possible that the relative lack of threats meant frequent vigilance was unnecessary; PGR has no large carnivores that may predate young elephants (e.g., lions) and other potential threats were common occurrences (e.g., vehicles and the train), and the elephants potentially habituated to these stimuli.

### 4.2. Cohesiveness

The association patterns of species exhibiting long-term relationships, including elephants, are known as fission–fusion dynamics [63]. Fission–fusion dynamics are characterised by the formation of temporary subunits and larger gatherings of multiple social groups, known in elephants as clans, that vary in size, composition, cohesion and duration within socio-ecological constraints.

Fission–fusion dynamics evolved due to intragroup competition for resources, ecological constraints [27], social needs [64,65], and (mis)aligned individual goals and motives [66]. In elephants, closely related females form strong bonds and tend to remain together for life. However, when socially disrupted and forced into groups of unrelated individuals (such as the Orphans), inclusive fitness benefits associated with elephants’ natural herd structure are negated, and one may expect that the individuals are less cohesive. Indeed, the Orphans proportionally fissioned more often than the A&B herd (Figure 4) and for longer periods of time (Figure 5a). Various subunits were formed during the observation times, which did not always include the same individuals, showing similarities to higher-order tiers in intact elephant herds [27].

Fission events are not only driven by sociality but also by ecological constraints. To determine the role of ecology in the fission–fusion dynamics of the Pongola elephants, we examined the seasonality of fissioning throughout the year. Ecologically, fissioning should occur most often during the dry season, when resources (food and water) are scarce and elephants fission to avoid competition with kin [27,67]. The dry season in PGR spans from June to August. The A&B herd is larger than the Orphans (Table 2), which might additionally mean competition amongst the A&B herd is comparatively more acute, and fission events are, therefore, more heavily influenced by seasonality. However, this was not the case. The Orphans fissioned more frequently during the autumn. The A&B herd, on the other hand, fissioned more often during the wet season (November–January). Potentially, the large size of the A&B herd meant that competition was a factor throughout the year and not so closely tied to seasonality, but still, the result was unexpected. Alternatively, the difficulty in keeping track of a large number of other elephants may have meant the A&B herd frequently fissioned for short periods of time during routine daily movements, though this does not explain the lack of fissioning during the dry season. Additionally, we must remember that the A&B herd were originally formed of two separate groups translocated from KNP and that one mature, dominant female was culled, events which may further contribute to the observed fission–fusion dynamics.

Fusion events help to form and maintain social relationships and exchange knowledge between herds. Upon first arriving at PGR, the Orphans (aged 10–13 years old at the time) attempted to assimilate with the A&B herd. We believe they were seeking the guidance of older females, which are known to act as repositories of social and ecological information [5,26] and possibly the security of a larger herd. However, it is rare that close-knit elephant herds admit non-related individuals [17,21,49], and this was the case for the Orphans.

### 4.3. Affiliative Interactions

We expected the reduced cohesiveness of the Orphans to additionally play out in the frequency of affiliative behaviours between herd mates, which is important for the formation and maintenance of relationships [68,69,70,71]. Contrary to expectations, however, the Orphans spent more time exhibiting affiliative behaviour relative to group size than the A&B herd. Further, the Orphans directed a greater proportion of their affiliative behaviours towards herd mates (other adult females and juveniles) as opposed to independent males. One explanation could be that the unrelated Orphans had to reaffirm their bonds and friendly intentions more often to ensure cohesion [71]. Alternatively, affiliative behaviour can also be a reaction to distress [72], and the early life experiences of the Orphans may have ingrained a greater dependence on affiliative behaviours in response. The larger proportion of affiliative behaviour expressed towards calves may, in particular, indicate uncertainty on the part of the Orphans’ adult females in calf-rearing, having not witnessed their own mothers and allomothers raising offspring.

The large proportion of affiliative behaviour directed from the A&B herd towards independent adult males may be a result of the greater number of females in the A&B herd; more females mean more individuals cycling through oestrous, during which time males preferentially associate with female herds. In addition, many of the adult males were born into the A&B herd and so may have engaged in affiliative behaviours to re-establish bonds with their natal herd.

### 4.4. Accelerated Reproduction

On average, African elephants give birth every 3.5–4.5 years, depending on ecological and climatic conditions [73,74,75,76]; however, orphaned elephants on other reserves have displayed rapid population growth due to shorter IBIs [77]. As hypothesised, the Orphans had significantly shorter IBIs than the A&B herd and, further, had shorter intervals than those recorded in KNP, where the Orphans originated from (3.65–4.0 years) [78]. Several factors could cause shorter IBIs. For instance, the death of a young calf would cause the mother to begin oestrous cycling again, thus shortening the normal IBI. However, calf death alone would not explain all shorter intervals, as the absolute infant mortality rate amongst the Orphans was not significantly different from the A&B herd. Early cessation of lactation and subsequent resumption of oestrus cycling may offer an alternative explanation, but an early cessation of lactation for a living calf would only occur under severe environmental conditions, which would likely also prevent conception and lengthen IBIs [56,79].

The age of first parturition did not significantly differ between the A&B herd and the Orphans, and it is notable that individuals in both herds had a very young age of first parturition at age seven. In the Orphans, Charlie (female, born in 2002) gave birth to her first calf, Isipho (male, born in 2009), at seven years old, meaning she reached sexual maturity and entered oestrous around age five. The same is true of Amethyst (female, born in 2004), who gave birth to her first calf at age seven (unknown, born in 2011). In non-disrupted wild elephant populations, the earliest known age of first parturition in the Amboseli National Park was just under nine years old [80], and in the Addo National Park population, the youngest recorded parturition was 10 years old [81]. An endocrinology study on 43 African elephants in European zoos found that the average age of sexual maturity was seven years, and the youngest known first parturition was eight years old (Ramat Gan Zoo) [82]. The age of the mother seems to have consequences on the health, survival, and reproduction of the offspring [83], whereby transgenerational effects are still poorly understood [34].

We believe the most likely explanation for the demographic rates seen in both herds, and particularly the Orphans, is that social disruption stimulates early maturation and reproductive acceleration [45,46,47,48]. This follows the concept of predictive adaptive response (PAR), by which conditions experienced in early life influence development. In humans, adverse conditions experienced in early life have been found to reduce life expectancy and decrease the age of sexual maturity [84]. Further, populations of small rodents rebound to pre-collapse levels at an accelerated rate [85].

In elephants, populations in the Tarangire and Gorongosa National Parks experienced accelerated rates of reproduction following heavy poaching [23,86], and individuals who lost their mother before the age of nine years in the Amboseli reached reproductive maturity earlier, although only by a few months [80]. Additionally, anecdotal evidence from recipients of other KNP culling orphans reported that these elephants reproduced rapidly, with IBIs as short as two years or less.

### 4.5. Sex Ratios

We thought it also worth discussing the apparently skewed sex ratios of the Orphans (and, to a lesser extent, the A&B herd) as a potential demographic impact of social disruption. The Orphans had a male:female sex ratio of 22:1. Comparison with other socially disrupted populations across South Africa indicated that several other reserves also tend to show a bias towards male calves, with the exception of ex-trained elephants (Reserve 5, Figure 9) and a group which had been spilt by a fence by management to consist of 15 and 44 elephants, respectively, with the smaller group having only one male (Reserve 7a,b, Figure 9).

There is abundant evidence suggesting adults manipulate the sex ratio of their offspring [87], with several hypotheses as to why this is the case (for further details, see [87,88]). Most private smaller reserves in South Africa preferred not to introduce many males with the founding herd, but over the years, the sex ratio has evened to reflect a more natural distribution. As most private reserves have reached their limit in the number of elephants the fenced environment can sustain, there may be a resource or density factor (or a cost-benefit effect) influencing the sex at conception [58].

Due to the small sample sizes across the included reserves, we can only speculate at this stage, and it may be, given time and further births, that the sex ratios will move towards the expected 1:1 ratio. However, this may be interesting to study further, especially for elephants confined within fenced systems.

### 4.6. Decision-Making and the Role of Matriarchs

Coordination of behaviour within a group is beneficial to all involved but requires a group decision [89]. In elephant society, decisions are predominantly made by the matriarch [26,66,76], who is related to all individuals in her herd and thus has an inclusive fitness interest in keeping the group safe and healthy [20]. The matriarch is often the oldest female in a group, and other herd members benefit from her experience [5].

In socially disrupted groups of unrelated and similarly aged elephants, one female may well take on the role of matriarch, although she will not have the same genetic motivation as in a natural herd, allowing individuals to make alternative decisions, leading to the splitting of the group [90], as was seen in the Orphans. Dominance hierarchies amongst female elephants have been established [91]; however, rather than aggressive interaction between females, dominance is established through an individual’s capacity to influence the behaviour of others and is closely related to personality [66]. In the Orphans, Constant (female, born in 1987) assumed the role of matriarch. We believe her protectiveness of the herd and particularly of calves, along with her strong allomothering instincts, secured her the role.

Observations of several other young KNP orphan groups noted that the oldest female of the group often assumed the matriarch role, with the second-oldest female assisting in group defence [49]. However, this was not always the case. In one instance, a group of young orphans (2–4 years old) promoted the oldest male to the role of matriarch and implicitly followed him. In another case, although a slightly older female was present, she seemed incapable of assuming this role, and these elephants split into smaller groups. It may be that personality, or genetic disposition, facilitates the acceptance of taking on this demanding role. There is further evidence that variations in maternal care can serve as the basis for genetic [92] and nongenomic [93,94,95,96] behavioural variation in transmission across generations.

The role of the matriarch is demanding, and not any older female appears capable of taking this on, resulting in the entire herd suffering. A study [97] documented the events following the death of a matriarch in an orphaned group. The next oldest female was reluctant to take on the role of matriarch, and instead, the herd followed a male. Under his leadership, a calf and a separate female were killed by another female, and a young male caused significant damage to the infrastructure of the reserve. Subsequently, the group displayed more erratic movements and fissioned often.

The A&B herd was initially composed of two family herds separately translocated from KNP but soon formed a single herd under the leadership of a dominant adult female, Buga (a female born in 1963). Buga was a particularly high-strung individual, potentially as a result of her translocation, and was culled in 2011 after showing repeated aggressive behaviour towards vehicles. Antares (a female born in 1963) took on the role of matriarch in 2009, and the A&B herd grew under her leadership.

Both matriarchs (Antares and Constant, the A&B herd and Orphans, respectively) spent a significant portion of time in typical positions ascribed to matriarchs in relation to the rest of the herd (leading the herd from the front, moving on the outer edge of the herd, or following the herd [54]), although Constant spent more time in these positions. This may reflect the size of the two herds, with the A&B herd being significantly larger than the Orphans (Table 2) and therefore being more difficult for Antares to coordinate. Alternatively, due to the non-relatedness of the Orphans, perhaps Constant felt the need to assert her role as matriarch more often.

In terms of ecological decision-making, we found differences in the directional movement patterns of the A&B herd compared with the Orphans. This was clearly demonstrated by the move of both herds to RJ in 2016. The A&B herd decisively moved from PGR to PNR-WS and then to RJ, with very few back-and-forth movements. The Orphans, on the other hand, were more cautious, initially making the transition later than the A&B herd and then even moving back to PNR-WS and Dubula after first venturing into RJ. This indecision may demonstrate the Orphans’ matriarch’s nervousness in large-scale movement decisions, which would, in a natural herd, be guided by and supported by older females.

### 4.7. Effect of Translocation on the A&B Herd

The A&B herd originally consisted of two separate family groups translocated from KNP that subsequently fused under one matriarch. Several unexpected behaviour patterns indicated that the translocation of the A&B herd from a familiar social network within KNP may have affected them. The A&B herd lost several calves to drowning, potentially as a result of them spending more time in open grasslands near the lake’s edge or alternatively due to a lack of understanding of the danger of water. Furthermore, they did not fission as expected during the dry seasons [27,67], again suggesting a lack of knowledge. Of interest was the relatively early age of first parturition, similar to the Orphans and zoo elephants, but much earlier than undisturbed herds elsewhere (Amboseli and Addo National Parks). The A&B herd also demonstrated atypical demographics and behaviours, indicating that any translocation of elephants away from familiar surroundings and social networks, even as intact family herds, can have long-term implications for behaviour, reproduction, and possibly herd fitness.

Emerging evidence of animal culture across diverse taxa seems to indicate that social learning and culture interact with processes important to management [98]. While the culture of elephants has not been a priority research topic to date, studies on other species show the importance of including the cultural aspect in defining long-term behaviour [99,100,101,102]. As such, the translocation of small elephant family groups away from their natal social network is likely to have long-term effects on the animals concerned, with consequences for their conservation and management.

## 5. Conclusions

In social species such as elephants, ecological and social knowledge and skills are transmitted from one generation to the next through learning. Socially disrupted elephant groups have an altered learning environment that may result in the loss of social and ecological competence [2]. This could have serious implications on current and future generations, affecting their fitness, with eventual implications for management and conservation [97,101].

Our study compared the social and environmental competence of two elephant groups, the A&B herd and the Orphans, both socially disrupted by management interventions previously and currently employed in South Africa. This unique long-running, individual-based behavioural dataset provides a glimpse into the impacts of social disruption through translocation on elephants.

There were marked differences in the social and ecological competence of the A&B herd and the Orphans. In line with theories that social disruption stimulates reproductive acceleration [45,46,47,48], the Orphans experienced shorter IBIs than the A&B herd. However, both groups experienced earlier-than-expected first parturition, in line with the theories of early maturation due to social disruption [45,46,47,48]. Food for thought is the apparent male bias in sex ratios at birth, with similar trends in some other reserves, though clearly, further research with a much larger dataset is needed to draw any conclusions.

Whilst both groups exhibited fission–fusion dynamics, the Orphans fissioned more often and for longer periods of time than the A&B herd. Interestingly and contrary to expectations, the A&B herd infrequently fissioned during the dry season, suggesting that fissioning was not ecologically driven but determined by other factors.

Overall, social disruption, including the loss of family structure and role models and the loss of a larger established network, reduces opportunities to learn, resulting in diminished social and ecological competence. This, in turn, has implications for the management and conservation of the species. Severed societies may, in the long term, adapt to new situations, but a huge amount of acquired knowledge will be lost.

Translocation is emerging to have more, and longer-term unintended consequences than initially anticipated, depleting elephant populations of their optimised social abilities, strategies, and possibly even culture. Although translocation is still viewed as a favourable management tool for local population control, it should only be performed following careful considerations of the long-term consequences, benefits and impairments to conservation, and only by translocating large groups of several families in order to minimise the loss of social networks and culture.

## Figures and Tables

**Table 1:** PGR’s founding elephant population. Age classifications: adult ≥ 15 years, subadult ≥ 10 and <15 years, juvenile ≥ 2 and <10 years, and calf < 2 years.

Date	Origin	Numbers
9 June 1997	Translocated from KNP as an intact family group (A family).	Eight made up of: three adult females; two subadult females; two juvenile females; one male calf.
13 June 1997	Translocated from KNP as an intact family group (B family).	Nine made up of: two adult females; four subadult females; one juvenile female; one juvenile male; one male calf.
July 1998	Translocated from KNP.	Three adult males
March 2000	KNP culling orphans initially translocated to a nearby reserve but subsequently moved into PGR (the Orphans).	Five made up of: four subadult females; one subadult male.
August 2001	Initially translocated from KNP to a nearby reserve, but subsequently moved into PGR.	Three adult males

**Table 2:** Elephant group size and composition at the end of the study period (December 2018). Age classifications: adult ≥ 15 years, subadult ≥ 10 and <15 years, juvenile ≥ 2 and <10 years, and calf < 2 years.

Group	Age Classification	Numbers
A&B herd	Adult females	17
	Sub-adult females	5
	Sub-adult males	4
	Juveniles	21
	Calves	12
Orphans	Adult females	5
	Sub-adult females	0
	Sub-adult males	5
	Juveniles	11
	Calves	1
Adult males	Independent bulls	13
Total		94

## Data Availability

The data presented in this study are available upon reasonable request from the corresponding author. The data are not publicly due for privacy reasons.

## Supplementary Materials

The following supporting information can be downloaded at: https://www.mdpi.com/article/10.3390/ani13030483/s1, Table S1: Ethograms of behaviours used for activity budget data collection: 30 min scan data.
